# Identification of highly effective target genes for RNAi-mediated control of emerald ash borer, *Agrilus planipennis*

**DOI:** 10.1038/s41598-018-23216-6

**Published:** 2018-03-22

**Authors:** Thais B. Rodrigues, Jian J. Duan, Subba R. Palli, Lynne K. Rieske

**Affiliations:** 1 University of Kentucky, Department of Entomology, Lexington, 40546 USA; 2 USDA ARS Beneficial Insects Introduction Research Unit, Newark, Delaware USA

## Abstract

Recent study has shown that RNA interference (RNAi) is efficient in emerald ash borer (EAB), *Agrilus planipennis*, and that ingestion of double-stranded RNA (dsRNA) targeting specific genes causes gene silencing and mortality in neonates. Here, we report on the identification of highly effective target genes for RNAi-mediated control of EAB. We screened 13 candidate genes in neonate larvae and selected the most effective target genes for further investigation, including their effect on EAB adults and on a non-target organism, *Tribolium castaneum*. The two most efficient target genes selected, *hsp* (heat shock 70-kDa protein cognate 3) and *shi* (shibire), caused up to 90% mortality of larvae and adults. In EAB eggs, larvae, and adults, the *hsp* is expressed at higher levels when compared to that of *shi*. Ingestion of dsHSP and dsSHI caused mortality in both neonate larvae and adults. Administration of a mixture of both dsRNAs worked better than either dsRNA by itself. In contrast, injection of EAB.dsHSP and EAB.dsSHI did not cause mortality in *T*. *castaneum*. Thus, the two genes identified cause high mortality in the EAB with no apparent phenotype effects in a non-target organism, the red flour beetle, and could be used in RNAi-mediated control of this invasive pest.

## Introduction

RNA interference (RNAi) is a specific gene-silencing mechanism that is being developed as a tool in pest management programs to protect plants against insect pests. Because its mode of action involves silencing specific target genes based on complementary sequences, RNAi is considered more specific than broad-spectrum and conventional pesticides, decreasing the chances of collateral damage and negative effects on non-target and beneficial organisms^1^. In addition, the diversity of Dicer-substrate siRNAs produced after dsRNA cleavage may impede the evolution of RNAi resistance due to polymorphism in the nucleotide sequences, thereby increasing the durability of RNAi technology in the field^2^. Utilization of RNAi technology to protect agricultural crops is more advanced than in horticultural crops or forestry^3–6^.

The efficacy of RNAi varies among insects, and several critical factors may be responsible for the differences observed. The target gene(s) and the level of expression^7–9^, the concentration of double-stranded RNA (dsRNA) used^10^, combinations of different dsRNAs^11^, and delivery methods^12^ could affect RNAi efficacy. dsRNAs are delivered through transgenic plants or by topical applications including trunk injection, root absorption, or spraying^2^. Since deployment of genetically engineered trees in reforestation remains contentious^5,13^, non-transgenic RNAi methods may be more readily accepted by foresters, land managers, conservationists, and the general public.

RNAi works well in beetles^14,15^ and therefore, coleopteran forest pests including the emerald ash borer (*Agrilus planipennis*, EAB) and Asian longhorned beetle (*Anoplophora glabripennis*, ALB) are potential targets for RNAi-mediated control. Both beetles are non-native introductions that have become invasive in North America due to an abundance of highly susceptible host plant material and a lack of effective population regulators^16,17^. Both EAB and ALB have been shown to respond to RNAi, therefore this technology may be feasible for their control^11,18^.

Initial work with EAB demonstrated that oral delivery of dsRNA targeting *iap* (inhibitor of apoptosis) and *cop* (COPI coatomer, β subunit) genes causes mortality and gene silencing in neonate larvae after 10 days of exposure^11^. However, selection of optimal target gene(s) for a given insect requires extensive screening of multiple dsRNAs, which is typically done via micro-injection. In *Tribolium castaneum*, for instance, a large-scale screen identified 40 most effective genes that caused 50–100% mortality at 8 days post-injection^9^; eleven of those genes caused >80% mortality on day 6, and 100% on day 8 after injection.

Screening target genes for RNAi by microinjection of dsRNA is not an option for EAB neonate larvae because of their small size, delicate form, and their endophagous feeding habits^11^. Additionally, RNAi efficiency varies with delivery method, and dsRNA injection is typically more efficient than dsRNA feeding^19^. Finally, oral dsRNA delivery is essential given that one of our goals is to develop a method for controlling EAB using RNAi technology.

We first screened 13 genes by feeding dsRNA to neonate larvae and selected two target genes that caused the highest mortality. The expression of two selected genes was evaluated in EAB eggs, larvae, and adults, and knockdown of target genes after dsRNA exposure was determined in larvae and adults. Additionally, we evaluated the mortality rate after ingestion of individual and combined dsRNAs in both larvae and adults. Finally, a non-target organism, *T*. *castaneum*, was used to evaluate the specificity of the EAB.dsRNA we designed.

## Results

### Screening of candidate genes

**Figure 1:**
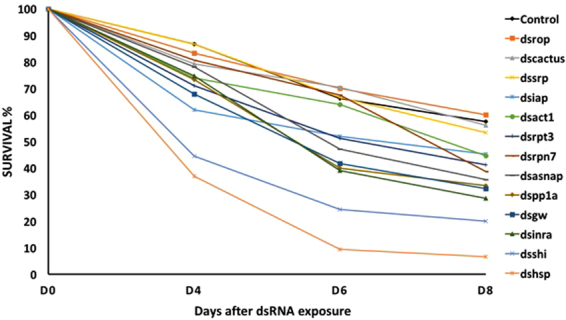
Screening of dsRNAs in EAB neonate larvae. Using a droplet feeding bioassay, neonate larvae were fed on dsRNAs at 10 µg/µL concentration for 4 d, followed by feeding sucrose solution without dsRNA. After day 4, mortality was recorded every 2 d until day 8. dsIAP was used as a positive control and dsmalE or dsGFP were negative controls. Bioassays were repeated twice for each treatment and 6x for control.

The mortality of neonate larvae following ingestion of dsRNA varied among the genes tested with most causing 13 to 38% mortality (Fig. 1). After 4 d of dsRNA exposure, 13.2% mortality was recorded in negative controls (dsGFP and dsMalE), and 38% mortality in our positive control (dsIAP). However, ingestion of dsRNA targeting *shi* and *hsp* caused 55.5 and 63% mortality after 4 d, and reached 80 and 93.3% mortality, respectively, after 8 d of dsRNA exposure. Among the 13 genes targeted, ingestion of dsSHI and dsHSP was most effective in EAB neonates, causing the highest mortality following dsRNA exposure.

### dsRNA dosage response

**Figure 2:**
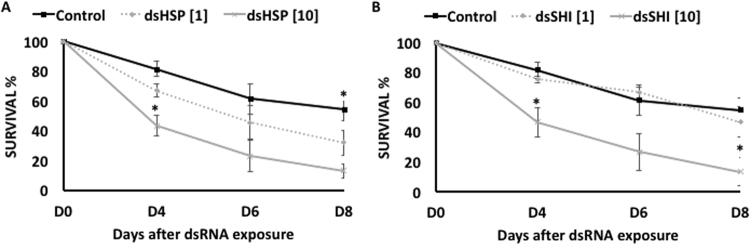
EAB survival is evaluated after 8 d of feeding on dsRNA. (**A**) Neonate larvae were fed on two different concentrations of dsHSP for 4 days. Control = dsmalE or dsGFP at a concentration of 10 µg/µL (N = 6); dsHSP [1] = 1 µg/µL (N = 4); dsHSP [10] = 10 µg/µL (N = 3). (**B**) Neonate larvae were fed on two different concentrations of dsSHI for 4 days. dsmalE or dsGFP at a concentration of 10 µg/µL (N = 6) were used as control. dsSHI [1] = 1 µg/µL (N = 3); dsSHI [10] = 10 µg/µL (N = 4). The asterisk reflects significantly different mortality rate (ANOVA, Student-Newman-Keuls, P = 0.002 (**A**), P = 0.01 (**B**)).

The two genes that caused the highest mortality, *hsp* and *shi*, were selected for further experiments to determine optimum dsRNA doses. Neonate larvae were fed on high (10 μg/μL of dsHSP or dsSHI) and low (1 μg/μL of dsHSP or dsSHI) doses of dsRNA. Neonates that ingested the high dose of either dsRNA experienced 90% mortality, whereas those that ingested low doses of dsHSP experienced 67% mortality after 8 d (Fig. 2A). In contrast, mortality of neonates ingesting 1 μg/μL of dsSHI did not differ from the control (Fig. 2B).

### Gene expression

**Figure 3:**
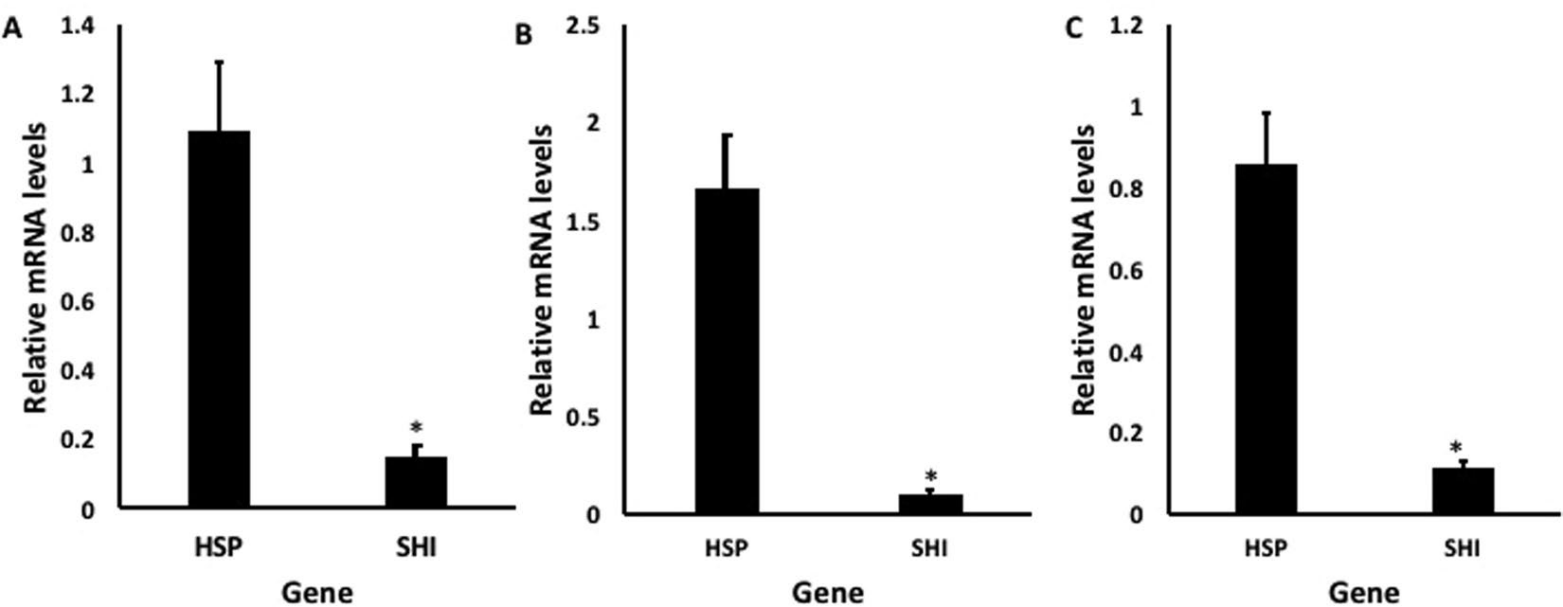
Relative expression of *hsp* and *shi* genes in EAB eggs, larvae, and adults. Eggs (**A**), neonate larvae (**B**) and adults (**C**) were exposed to dsGFP (control) and total RNA isolated after 48 h (**A**), 72 h (**B**), and 24 h (**C**). Relative mRNA levels were normalized using TEF as a reference gene. Mean + S.E (A: N = 3, B: N = 3; C: N = 5) are shown. The asterisk denotes significant differences (t-test, two-tailed P-value: P = 0.002 (**A**), P = 0.009 (**B**), P = 0.002 (**C**)).

The relative expressions of *hsp* and *shi* genes were compared in EAB eggs, larvae, and adults (Fig. 3A–C). In all life stages tested, *hsp* gene expression is >8x more than *shi* gene expression.

### Combination of dsRNA

**Figure 4:**
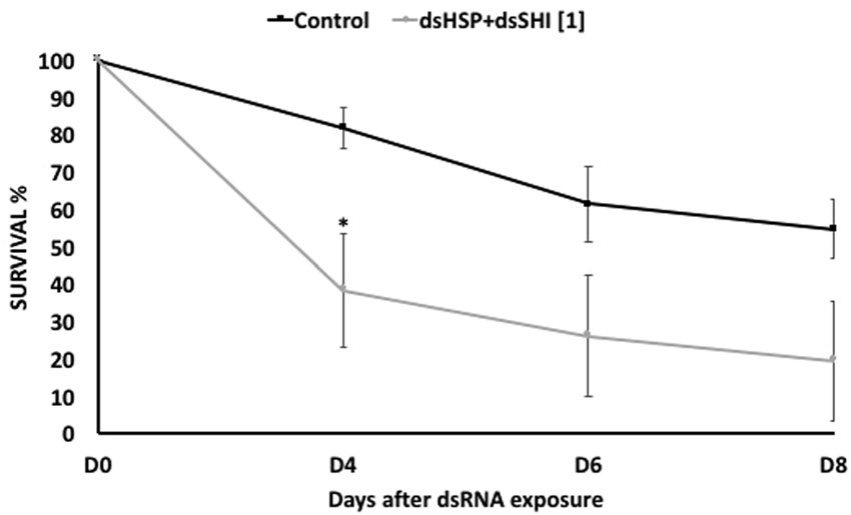
Larval EAB survival after 8 days fed on a combination of dsRNAs. Neonates were exposed to 1 µg/µL of dsRNA (500 ng/µL each dsHSP and dsSHI; N = 3) for 4 consecutive days and then fed on blue-sucrose solution without dsRNA until day 8; 10 µg/µL of dsmalE or dsGFP (N = 6) were used as control. The asterisk denotes days of exposure when treatments are significantly different (t-test, two-tailed P-value: P = 0.007).

Neonate larvae fed on 1 µg/µL of both dsHSP and dsSHI (500 ng/µL of each) for 4 d had higher mortality after 4, 6, and 8 d (61.6%, 73.8%, and 80.5%, respectively; Fig. 4) than the larvae fed on 1 µg/µL of dsHSP (Fig. 2A) or 1 µg/µL of dsSHI (Fig. 2B).

### Gene silencing

**Figure 5:**
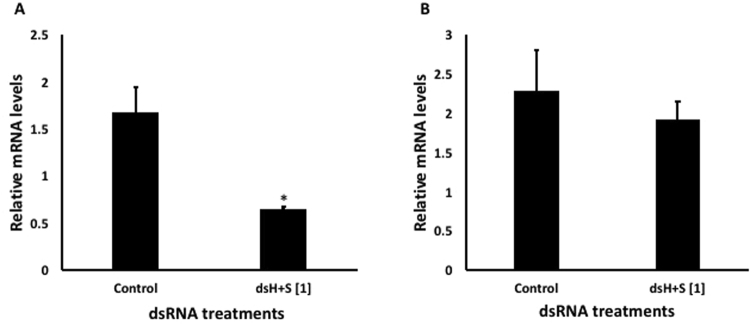
Relative expression of *hsp* (**A**) and *shi* (**B**) genes in EAB larvae after 3 days feeding on 1 µg/µL of combined dsRNAs (500 ng/µL each dsHSP and dsSHI); 10 µg/µL of dsGFP was used as control. Relative mRNA levels were normalized using TEF as a reference gene. Mean + S.E (N = 3) are shown. The asterisk above the bar indicates significantly different expression (t-test, two-tailed P-value: P = 0.038 (**A**), P = 0.635 (**B**)).

**Figure 6:**
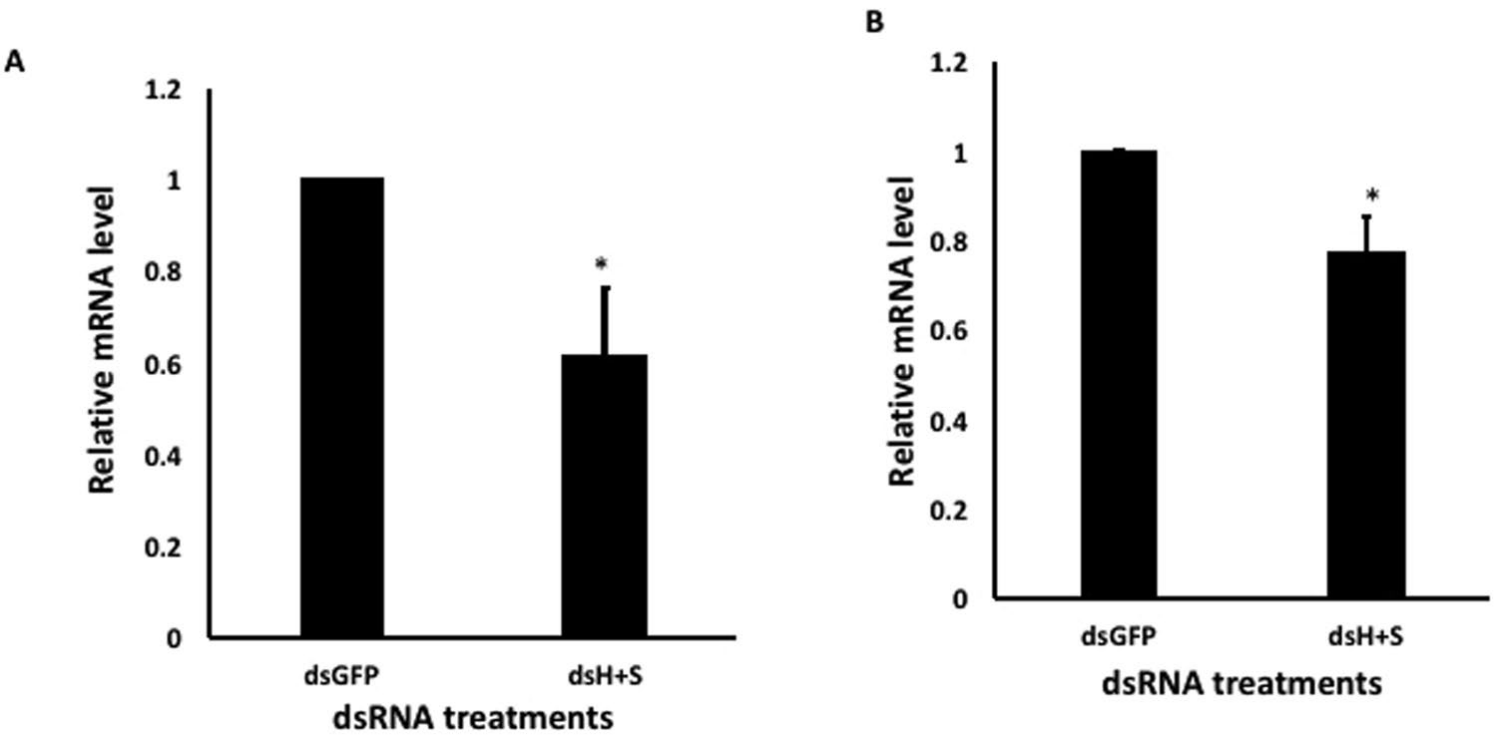
Relative expression of *hsp* (**A**) and *shi* (**B**) genes in EAB adults 24 h after injection of 20 µg/µL of dsRNA (10 µg/µL each dsHSP and dsSHI); 20 µg/µL of dsGFP were used as control. Relative mRNA levels were normalized using TEF as a reference gene. Mean + S.E (N = 5) are shown (t-test, two-tailed P-value: P = 0.05 (**A**), P = 0.02 (**B**)).

Neonates fed on 1 µg/µL of both dsHSP and dsSHI (500 ng/µL of each) for 3 d showed a decrease in the mRNA levels of *hsp* (62%) and *shi* (15%) when compared to their levels in neonates that fed on dsGFP (Fig. 5A,B). In adults, injection of 20 µg/µL of both dsHSP and dsSHI (10 µg/µL of each) resulted in 20–40% silencing (p ≤ 0.05) of both *hsp* and *shi* genes at 24 h after injection (Fig. 6A,B).

### Adult mortality

**Figure 7:**
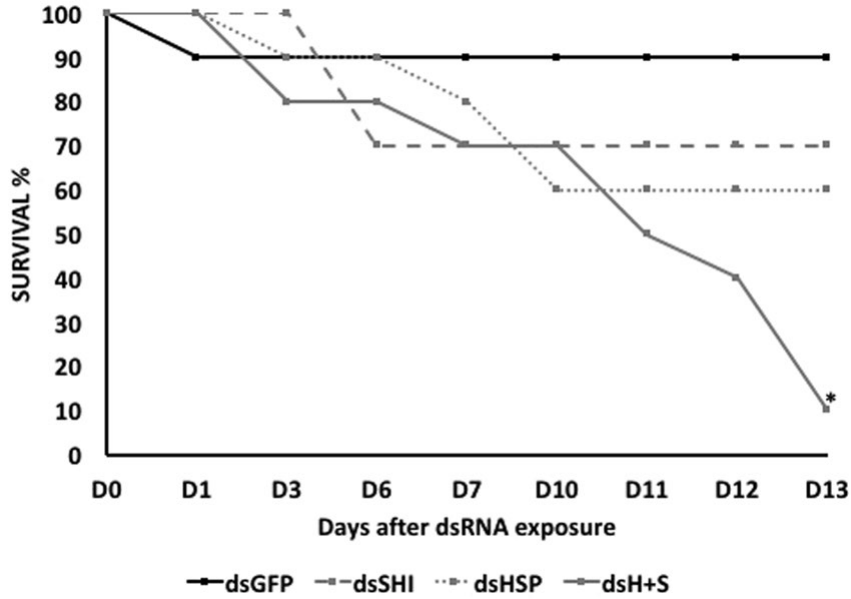
Adult EAB survival at 14 days after feeding on 2 µL of dsRNA at a final concentration of 10 µg/µL of single dsGFP (control), single dsHSP, and single dsSHI and 1 µg/µL of combined dsHSP and dsSHI (500 ng/µL of each). The asterisk indicates significant differences in mortality between treatment and control (Fisher’s Exact test, two-tailed, P-value = 0.011, N = 10).

In the adult feeding trial, beetles were fed on 10 µg/µL dsSHI, 10 µg/µL dsHSP, 1 µg/µL dsHSP + dsSHI (500 ng/µL of each), or a 10 µg/µL dsGFP control. There were no effects detected on day 1 with the exception of a single beetle lost in the control treatment, which then remained constant through day 13. Adult beetles fed on dsSHI experienced 30% mortality at 6 d, which persisted through the experiment. Adult mortality became evident at 3 d in adults fed on dsHSP (10%); mortality rose to 40% for dsHSP-exposed beetles for the duration of the experiment. However, for beetles fed on 1µg/µL of combined dsHSP and dsSHI, 90% mortality was evident on day 13 (Fig. 7).

### EAB.dsRNA in *Tribolium castaneum*

The nucleic acid sequences of the EAB *hsp* and *shi* showed 85% and 91% identity, respectively, with the homologues in *T*. *castaneum*. Injection of EAB dsHSP and dsSHI into *T*. *castaneum* adults caused no mortality (Table 1).

**Table 1:** Similarity between *A*. *planipennis* dsRNA designed sequences and *T*. *castaneum* genes.

Gene Name	*T*. *castaneum* Accession	Query Cover	E-value	Identity	Mortality* (10 dai)
*shi*	XM_008200600.2	92%	3e-49	91%	0%
*hsp*	XM_015982882.1	78%	7e-29	85%	0%

dai: days after dsRNA injection. *Sun-Shepard’s formula was used to adjust for mortality.

## Discussion

Adoption of RNAi into management strategies against forest pests lags behind that of agricultural pests, but numerous native and non-native forest insect pests are appropriate candidates for this technology. Emerald ash borer is a non-native, phloem-feeding beetle that has killed millions of trees in North America; its invasion is unprecedented in scope and magnitude^17^. Although chemical suppression is possible in managed settings^20^ and classical biological control has been implemented with considerable success^21,22^, the invaded range continues to expand and ash trees continue to die. The need for innovative approaches to EAB management remains high^17^.

We are investigating the feasilibility of utilizing RNAi as a means of suppressing EAB, and previously demonstrated that EAB has efficient RNAi machinery, and that neonates ingesting dsIAP and dsCOP, targeting *iap* and *cop* genes, experienced significant mortality^11^. However, selection of target genes with rapid action is essential, as is an oral method to deliver dsRNA molecules to EAB. Here, we used a neonate larval feeding assay to screen 13 target genes, and selected two that are especially efficacious and demonstrate potential for incorporation into an EAB management scheme.

A significant mortality was observed 4 to 8 days after dsRNA ingestion, depending on dsRNA concentration and target gene, corroborating data reported previously in *T*. *castaneum*^9^. At 8 d after dsRNA ingestion, the effect of the dsRNAs causing EAB neonate mortality clustered into three groups (Fig. 1). The first group has three genes that caused 40–47% mortality and did not differ from the effects observed in the control treatment (45%). The second group of eight genes caused 55–72% mortality and included the positive control dsIAP. However, the third group included the genes *shi* and *hsp*, which caused 80% and 93% mortality, respectively. Both *shi* and *hsp* caused significant mortality after 4 and 6 d following ingestion of dsRNAs targeting these specific genes.

We then evaluated the effects of these selected dsRNAs in two different concentrations. In studies using *Bactrocera dorsalis* (Diptera: Tephritidae), the concentration of dsRNA influenced the level of gene suppression^23^, and in studies using oral delivery of dsRNA to *Diabrotica v*. *virgifera* and *A*. *planipennis*, there was a positive correlation between dsRNA concentration and mortality rate^11,24^. The most efficient mortality response in EAB was observed on the 8th day after ingestion of the highest concentration of dsHSP and dsSHI (~90%; Fig. 2A,B). Ingestion of the lower concentration of dsHSP alone also caused a significant larval mortality (67%), but the lower concentration of dsSHI alone (53% mortality) did not differ from the control (45%). One explanation for differences in efficiency among target genes may be attributed to the relative expression profile of the target genes. In EAB eggs, neonate larvae, and adults, the *hsp* gene is highly expressed relative to *shi* (Fig. 3). Highly expressed genes are considered more susceptible to RNAi gene silencing responses, suggesting that the abundance of target mRNAs is one of the critical factors that determine the efficiency of the RNAi in a given cellular context^7^. Corroborating this, we also found that the relative silencing of *hsp* was greater than *shi* silencing (62% versus 15%) (Fig. 5). It is interesting that ingestion of a combination of two dsRNAs at low concentrations (500 ng of each dsSHI and dsHSP) by neonates and adults efficiently induced mortality. Exposing neonates to dsSHI + dsHSP, in a final concentration of 1 μg/μL caused 62% mortality in 4 days (Fig. 4), whereas, dsHSP alone caused 33%, and dsSHI alone caused 25% mortality (Fig. 2A,B, respectively).

Even larvae that were exposed to a 10x higher concentration of single dsRNA treatment showed lower mortality than that observed after treatment with both the dsRNAs, and similar results were observed in adults. After 2 weeks, adults fed with combined dsRNA in 10x lower concentration experienced 90% mortality (Fig. 7). On the other hand, when the insects were fed on a single dsRNA in high concentration, 30–40% of mortality was observed. Although competition among multiple dsRNA’s can potentially confound RNAi efficiency^25^, our findings corroborate previous studies that showed additive effects of combined dsRNA^9,11^ in EAB. As a target, *shi* appears to be less efficient, but when supplemented with *hsp*, *shi* may become more potent. However, further studies on gene expression patterns and other combinations of genes must be performed to better understand and confirm these findings.

These two genes, *shi* and *hsp*, were silenced after dsRNA injection in *T*. *castaneum* adults, where they caused 100% of mortality^9^. In our study, after injecting *T*. *castaneum* with dsHSP and dsSHI designed specifically to silence EAB genes, no mortality was observed after 10 days. Our results suggest that EAB.dsSHI and EAB.dsHSP appear specific to our target insect and appear to have negligible effects in a non-target organism. However, bioassays must be performed to confirm the RNAi effects in other non-target organisms, especially the classical biological control agents *Tetrastichus planipennisi* (Eulophidae), *Spathius agrili* and *S*. *galinae* (Braconidae), and *Oobius agrili* (Encyrtidae), that form the mainstay of sustainable EAB management programs in North America^21^. Studies must also be performed to evaluate its safety for other off-target organisms, as well as dsRNA stability, transport inside the plant, and its fate in the environment^1^. Finally, development of applied systems to deliver dsRNAs remains to be developed. Encapsulated dsRNA in nanoparticles to be used as a foliar and trunk spray, soil treatment and root absorption, and tree trunk injections are some of the topical applications; genetically engineered trees expressing dsRNA are another possibility^2,26,27^.

Our findings suggest that *hsp* and *shi* are potential target genes to suppress EAB populations. As EAB continues its invasion of North America, we continue to accumulate additional non-native species, and endemic pests undergo extensive and destructive population fluctuations, we must increase our efforts to develop innovative approaches for forest pest management. Our work demonstrates that RNAi may become a feasible approach to suppressing pests in forests.

## Methods

### Insects

Laboratory-reared *A*. *planipennis* eggs were placed in Petri dishes (150 × 15 mm) with moistened filter paper and maintained at 23 °C and 75% relative humidity in a growth chamber. Newly hatched unfed neonate larvae at <48 h post-hatch were used in bioassays. Adults were reared in the laboratory at 23 °C from field-collected green ash (*F*. *pennsylvanica*), and maintained on either green or tropical ash (*F*. *uhdei*) foliage.

### Selection of target genes and synthesis of dsRNA

PCR templates for *in vitro* transcription of dsRNA were generated using gene-specific primers containing T7 polymerase promoter sequence (TAATACGACTCACTATAGGG) at the 5’end (Table 2). Candidate genes were selected based on previous reports of mortality in *T*. *castaneum* exposed to dsRNAs targeting these genes^9^. PCR conditions were 94 °C for 4 min, followed by 35 cycles of 94 °C for 30 s, 60 °C for 30 s and 72 °C for 45 s, finishing with an extension step at 72 °C for 10 min. The PCR template was purified using a PCR purification kit (Qiagen Inc., Valencia, CA USA). As a negative control, a fragment of GFP (green fluorescence protein) was used. After PCR purification, dsRNA synthesis was performed using the MEGAscript RNAi Kit (Ambion Inc., Foster City, CA USA) following manufacturer’s instructions. Briefly, 200 ng of purified PCR product was used as template in a 20 μL *in vitro* transcription reaction. The reaction was incubated for 16 h at 37 °C, followed by 15 min of DNase treatment. The dsRNA was precipitated by adding 0.1x volume of sodium acetate (3 M, pH 5.2) and 2.5x the volume of 100% ethanol; kept at −20 °C for at least 2 h followed by centrifugation at 4 °C for 30 min. The dsRNA pellet was then rinsed with 750 μL of 75% ethanol and centrifuged again at 4 °C for 15 min. The ethanol was removed and the dsRNA was diluted in ultrapure distilled water. The quality of the dsRNA was checked by electrophoresis and quantified using a spectrophotometer (NanoDrop Technologies, Wilmington, DE USA). When a higher concentration of dsRNA was needed, the samples were vacuum concentrated using Concentrator plus (Eppendorf, Hauppauge, NY, USA).

**Table 2:** Primer sequence, dsRNA size and NCBI sequence ID for the target genes. All primers contain the promoter sequence of T7 RNA Polymerase that is not represented in the table.

Gene Name	Primer name-dsRNA size	Primer Sequence	Ap sequence ID
srp54k	ds - EAB srp - 496 bp – F	GGCTGTAGCAAATGCAGTGA	XM_018478973.1
	ds - EAB srp - 496 bp - R	ACGCGCCATAGACTCTTGTT	
ras opposite	ds - EAB rop - 493 bp – F	GCCGACACCTTTCAGTGTTT	XM_018464595.1
	ds - EAB rop - 493 bp - R	GCTTGGAATCGGTGAACTTT	
shibire	ds - EAB shi -483 bp – F	TGGCACATTTGTATGCCAGT	XM_018465318.1
	ds - EAB shi -483 bp - R	CTTGTTGCATTTGCTGAGGA	
protein phosphatase 1 alpha at 96a	ds - EAB pp1a - 532 bp – F	TATGTGTACGTGTGCCCGTT	XM_018468028.1
	ds - EAB pp1a - 532 bp - R	TTGATGAAGAGCAAGCGAAA	
regulatory particle non-atpase 7	ds - EAB rpn7 - bp – F	TTGAAGAGGGAGGTGATTGG	XM_018468408.1
	ds - EAB rpn7 - bp - R	TGATCCGGCCTATTTGTCTC	
regulatory particle triple-a atpase 3	ds - EAB rpt3 - 513 bp – F	GCCAAAGCAGTAGCACATCA	XM_018479000.1
	ds - EAB rpt3 - 513 bp - R	AGCATGCATTCCAGCTTCTT	
actin	ds - EAB act1 - 427 bp – F	GCTAACCGCGAGAAGATGAC	XM_018481271.1
	ds - EAB act1 - 427 bp - R	GGAACCTTTCGTTTCCAACA	
cactus	ds - EAB cact - 389 bp – F	ATGTTGTGTTGGTGCGAAAA	XM_018478973.1
	ds - EAB cact - 389 bp - R	TTCCCGAACTAAGGTCGTTG	
alpha snap	ds - EAB asnap - 484 bp – F	GTAGTGCATTTTGCGAAGCA	XM_018465131.1
	ds - EAB asnap - 484 bp - R	TGACGAGCATTCAGCAAATC	
inverse regulator a	ds - EAB inra - 449 bp – F	ACCGGTGTTACCAAAGCAAG	XM_018464445.1
	ds - EAB inra - 449 bp - R	GCGCTATTAACAGGCGCTAC	
heat shock 70-kDa protein cognate 3	ds - EAB hsc70-3 - 468 bp – F	GTTACGAGCCAGGGTGAAAA	XM_018474521.1
	ds - EAB hsc70-3 - 468 bp - R	CTTTTGAACGGCACGGTTAT	
gawky	ds - EAB gw - 442 bp - F	CAACATTGCGCCGACTACTA	XM_018475118.1
	ds - EAB gw - 442 bp - R	CCACATTCCTCCTCCACTGT	

### Screening assays

A droplet bioassay^11^ was used to screen potential target genes for EAB suppression. Twelve candidate genes (Table 2) and positive and negative controls were evaluated. Inhibitor of apoptosis gene (*iap 1*), previously shown to cause mortality after dsIAP exposure in both EAB and ALB^11,18^, was used as a positive control, and green fluorescent protein (*gfp*) was used as a negative control. Droplets of dsRNA (2 μl each) at a concentration of 10 μg/μl diluted in blue sucrose solution were used to feed 3–4 neonate EAB larvae in covered petri dishes with moistened filter paper. The blue dye in the sucrose-dsRNA solution allowed us to confirm neonate larval consumption. Neonates were exposed to dsRNA for 4 consecutive days; droplets were replenished on the rare occasions when the solution was completely consumed or evaporated. On day 5, larvae were transferred to new plates containing droplets of sucrose solution without dsRNA. Larval mortality was recorded at 48 h intervals through day 8 after dsRNA feeding treatment. Experiments were repeated twice under the same conditions using 20 neonate larvae per treatment. If the two repeats showed variability, a third repeat was performed. The dsRNAs that showed higher mortality after 8 d were repeated at least one more time, for a total of three repeats; those with the highest EAB mortality were selected for further experiments to confirm their potential for use in managing EAB.

### Dosage response

Two concentrations, 1 μg/μL and 10 μg/μL, of dsHSP (targeting heat shock 70-kDa protein cognate 3 gene) and dsSHI (targeting shibire gene) were used to evaluate neonate mortality, and 10 μg/μL dsGFP was used as a control. Larvae were fed on dsRNA for 4 d, followed by 4 d on blue sucrose solution without dsRNA. Mortality (%) was then calculated based on the initial number of larvae (15–20) on day one. The experiment was repeated 3–6 times, and a one-way analysis of variance (ANOVA) was used with Student-Newman-Keuls to evaluate significance of differences.

### Combinations of dsRNA

The two selected dsRNAs, dsHSP and dsSHI, were combined to evaluate larval mortality and gene silencing using the droplet assay. The dsRNAs were diluted in sucrose solution to a final concentration of 10x lower single dsRNA (1 μg/μL dsSHI + dsHSP: 0.5 μg/μL of dsSHI and 0.5 μg/μL of dsHSP). The dsGFP at a concentration of 10 μg/μL was used as a control. Four biological replicates, with 15–20 neonate larvae per replicate, were used and a one-way ANOVA was used for data analysis, with Student-Newman-Keuls to detect significance of differences.

### Gene expression

Both *hsp* and *shi* genes were analyzed for gene expression patterns in eggs, larvae, and adults, and for knockdown after dsRNA exposure in neonate larvae and adults, using dsGFP as a control. For gene expression patterns, the control treatment in neonate larvae and adults was used for the analyses. The eggs were treated with 1 μg/μL of dsGFP followed by a 1 μL droplet of dsRNA (3 times during 48 h). Eggs were maintained at 28 °C for 48 h, and each treatment was performed in triplicate with 6–7 eggs per replicate. For gene knockdown analyses, neonate larvae were fed on both dsSHI and dsHSP (1 μg/μL total, 0.5 μg/μL of each dsRNA) for 72 h; 10 μg/μL of dsGFP was used as a control. Larval assays were performed in triplicate with 6–8 neonates per replicate. Adult beetles were evaluated for *hsp* and *shi* gene silencing 24 h after injection with 2 μL of both dsRNAs (dsSHI and dsHSP at 20 μg/insect total, 10 μg/μL of each dsRNA), using dsGFP (20 μg/insect) as a control. Micro-injection of dsRNA was chosen because it is faster, easier, and requires fewer insects to perform. Five biological replicates were performed using one beetle in each replicate. Following assays, eggs and neonate larvae were pooled for RNA extraction. RNA was also isolated from each adult beetle. A two-tailed t-test was used for statistical analysis to compare the means of a single variable.

### RT-qPCR

Total RNA was isolated from 6–10 EAB larvae pooled after feeding on dsRNA using the TRI Reagent RT (Molecular Research Center Inc., Cincinnati, OH, USA). The cDNA was synthesized using M-MLV Reverse Transcriptase (Life Technologies, Carlsbad, CA, USA) from RNA and used as a template for gene expression studies. The expression analyses of the target genes were conducted using SYBR Green PCR Master Mix. Briefly, the PCR mixture contained 1 μL of synthesized cDNA, 0.2 μL of each primer (10 mM; Table 3), 5 μL of the SYBR green PCR master mix and 3.6 μL of ddH_2_O. The reactions were carried out in duplicate per template in a final volume of 10 μL. RT-qPCR reactions were performed by the StepOnePlus Real-Time PCR system (Life Technologies, Carlsbad, CA, USA) using the following cycling conditions: one cycle at 95 °C (20 s), followed by 40 cycles of denaturation at 95 °C (3 s), annealing and extension at 60 °C for 30 s. At the end of each RT-qPCR reaction, a melting curve was generated to confirm a single peak and rule out the possibility of primer-dimer and non-specific product formation. The TEF1A was used as reference gene^28^, and 2^−ΔΔCt^ method was used to calculate the relative expression levels of the target gene in the samples compared to controls^29^. Standard curves were performed for all the new primers and the correlation coefficients and amplification efficiency parameters were analyzed. A two-tailed t-test was used for statistical analysis to compare the means of a single variable.

**Table 3:** Primer sequence and amplicon size for the target genes *shi* and *hsp*, for qPCR.

Primer Name	Primer Sequence (5′- 3′)	Amplicon (bp)
q- EAB shi - 122 bp - F	GGGATCTGCCCAAATTAACA	122
q - EAB shi - 122 bp - R	CCCGTCTGAGTTCTTTCTCG	
q- EAB hsp70-3 - 97 bp - F	GACAAAGGAACGGGAAACAA	97
q - EAB hsp70-3 - 97 bp - R	TCTCGGCATCCCTTATCATC	

### Adult mortality

A feeding assay was performed to deliver dsRNAs to EAB adults to evaluate the effects of silencing *hsp* and *shi* genes. The dsRNAs were diluted with 40% sucrose solution, and each beetle was fed on ~2 µL  droplet of the diluted solution of each dsRNA sample. Beetles were fed 10 µL of each dsRNA (dsGFP, dsHSP, and dsSHI), and 1 µL of the combination of dsHSP and dsSHI (500 ng of each). In each treatment, 5 male and 5 female beetles (N = 10) were evaluated. Mortality was recorded at 24–48 h intervals for 13 d. After the single dsRNA feeding exposure at day 0, adult beetles were provided with fresh tropical ash foliage and were kept in an environmental chamber (Percival Scientific, Perry, IA) at 25 + 1.5 °C, 55–65% RH, and a photoperiod of 16:8 (L:D) h. For data analysis, males and females were combined, and Fisher’s test was used to identify statistical differences between treatments.

### EAB dsRNA effect on *Tribolium castaneum*

To evaluate potential off-target effects of the EAB-specific dsRNA, both EAB.dsHSP and EAB.dsSHI or dsGFP as a control were injected into *T*. *castaneum* adults. For microinjections, 10–18 adults were anesthetized on ice and temporarily fixed on a glass slide using double-sided tape. 0.1 μL of 5 μg/μL dsRNA (0.5 μg/adult) was injected into the dorsal side of its first or second abdominal segment using Nanoject III Programmable Nanoliter Injector (Drummond Scientific Company, Broomall, PA, USA). After dsRNA injections insects were place in 12-well plates with wheat flour and held in darkness at 26 °C and 36% humidity. Insect mortality was recorded 10 d post-dsRNA injection. Sun-Shepard’s formula^30^ was used to adjust for mortality of controls. Bioinformatic analyses were performed to compare the sequences of gene fragment used for dsRNA synthesis from EAB and *T*. *castaneum*. Sequences of *hsp* and *shi* gene regions used for dsRNA synthesis from EAB were aligned to *T*. *castaneum* genes using MUSCLE software.
